# The impact of Mayer–Rokitansky–Küster–Hauser Syndrome on Psychology, Quality of Life, and Sexual Life of Patients: A Systematic Review

**DOI:** 10.3390/children9040484

**Published:** 2022-04-01

**Authors:** Ermioni Tsarna, Anna Eleftheriades, Makarios Eleftheriades, Emmanouil Kalampokas, Maria-Konstantina Liakopoulou, Panagiotis Christopoulos

**Affiliations:** 1 2nd Department of Obstetrics and Gynecology, Medical School, National and Kapodistrian University of Athens, 11528 Athens, Greece; makarios@hotmail.co.uk (M.E.); m.kalampokas@gmail.com (E.K.); m.liakopoulou@med.uoa.gr (M.-K.L.); panchrist@med.uoa.gr (P.C.); 2 Postgraduate Programme “Fetal Maternal Medicine”, Medical School, National and Kapodistrian University of Athens, 11527 Athens, Greece; annielefth-28@hotmail.com

**Keywords:** Mayer–Rokitansky–Küster–Hauser syndrome, adolescence, stress, mental health

## Abstract

Background: Mayer–Rokitansky–Küster–Hauser (MRKH) syndrome is a rare congenital syndrome characterized by uterovaginal agenesis. Most patients are diagnosed during adolescence, when body image and sexual identity are shaped. Our main objective was to investigate how MRKH syndrome affects psychology, quality of life (QoL), and the sexual life of patients compared with non-affected individuals. Methods: Original peer-reviewed research papers examining psychological outcomes, QoL, and sexual function of MRKH patients were searched in PubMed. Titles, abstracts, and full text from potentially eligible records were reviewed by two independent reviewers. Case reports and papers published not in English were excluded. Results: Our search identified 63 records, of which 20 were included: 10 examined psychological and psychosocial outcomes, 14 examined sexual function outcomes, and 6 examined QoL outcomes. Results may be affected by selection bias and confounding due to differences between MRKH patients and controls. Conclusions: MRKH could be associated with a higher prevalence of anxiety and depression symptoms and social insecurity compared with women of a similar age without the condition. MRKH could also be associated with greater pain and discomfort during sexual intercourse and limitations in arousal, lubrication, and orgasm. MRKH patients more commonly experience impairment of mental-health-related QoL, but physical-health-related QoL is not affected.

## 1. Introduction

Mayer–Rokitansky–Küster–Hauser (MRKH) syndrome is a congenital disorder of the female reproductive system estimated to affect 1 in approximately 5000 females; it is characterized by the absence of the upper part of the vagina with variable uterine development [1,2]. With regard to embryology, MRKH syndrome is regarded as a result of agenesis or hypoplasia of the Müllerian duct system, or a non-fusion of the Müllerian ducts with the Wolffian ducts [3]. Instead of an anatomically and functionally typical uterus, patients with MRKH syndrome might have an obstructed uterus, one or two rudimentary uterine horns, or cavitated Müllerian remnants; a functional endometrium might be present within the aforementioned underdeveloped uterus. Ovaries are functional in the majority of MRKH patients, resulting in normal secondary sexual characteristics, although hypoplastic ovaries have been described in a portion of MRKH patients [4]. In addition to the anatomic malformations of the reproductive system, many MRKH patients have associated anomalies of the urinary tract, skeletal anomalies, and less often other anomalies involving among others the heart, the cleft palate, and hearing ability.

Apart from the anatomical and functional anomalies, it is generally assumed that MRKH syndrome burdens those affected in terms of psychology, quality of life, and sexual function; difficulties in sexual intercourse, experience of shame due to the therapeutic interventions, and infertility are thought to contribute [5]. Several studies have examined the improvement of psychological status, quality of life, and sexual function in patients after some treatment has been performed, and direct comparison of different therapeutic interventions in terms of psychology, sexual function, and quality of life is also available [6]. However, less is known about the impact of MRKH diagnosis at adolescent girls before any therapeutic intervention, as well as the long-lasting impact of MRKH syndrome after any therapeutic intervention in comparison with women without the syndrome. Therefore, it remains unclear whether MRKH patients can expect a satisfying sexual life and a quality of life, comparable to the general population after the treatment, or whether a long-lasting impact of MRKH on patients’ psychology should be expected and managed appropriately.

The aim of this study is to systematically review the published research papers that examine the impact of MRKH syndrome on psychology, quality of life, and sexual life of the patients, both before and after different therapeutic interventions, as compared with non-affected individuals.

## 2. Materials and Methods

In this systematic review, we included original peer-reviewed research papers that examined psychological and/or psychosocial outcomes, quality of life, sexual function, and/or wellbeing of patients with MRKH syndrome, either before or after therapeutic interventions, compared with controls without MRKH syndrome. With regard to study design, randomized control trials (RCTs), cohort studies, case–control studies, and case series were considered as eligible in this review, as long as a direct comparison with a control group, reference sample, or the general population was performed and reported. Case reports and animal studies were excluded from this systematic review. Original research papers, which presented results on relevant outcomes for groups of patients with several diagnoses including MRKH, were included in this systematic review only if results were reported separately for the subgroup of MRKH patients. Research papers published in language other than English were not included in this systematic review.

PubMed was searched for potentially eligible research papers from inception to 8 January 2022. Our search algorithm included multiple terms that describe the MRKH syndrome as well as terms that describe the outcomes of interest for this review. A filter was applied to safely remove all animal studies, as well as studies for which the abstract was not available or there was no link for the full text. The search algorithm is presented in detail in Table 1.

All records retrieved from our search were screened by two independent reviewers. Only the records that clearly did not meet our inclusion and exclusion criteria were excluded at this phase. In case of discrepancy, the record was not excluded but rather assessed in full text. Full text from potentially eligible records was retrieved and reviewed by two independent reviewers. In case of discrepancy, a third reviewer additionally assessed the study’s full text for eligibility. Lastly, the reference list of all included research papers, as well as any excluded reviews, were manually searched for potentially eligible research papers that were not included in the results of our search algorithm.

Two independent reviewers tabulated the relevant data from the included research papers. Namely, we recorded the following factors: the year of publication; the county that the study took place; the recruitment strategy; the number of MRKH patients that were included in the study; details on the sample size and characteristics of the control group; the year of patients’ assessment and their age at assessment; the kind of treatment that has preceded, if any; the period that passed from treatment until patient assessment; finally, the outcomes that were examined in the study, the scales used, and the findings of the study with regard to these outcomes. In cases where comparisons between MRKH patients and controls were performed in multiple time points, all results were tabulated and are discussed in this systematic review. The tabulated data by the two independent reviewers were compared for discrepancies, in which case the research paper was read by a third reviewer that independently tabulated the aforementioned relevant data and agreement was reached via discussion between all three reviewers.

To summarize and synthesize the results of the included studies, we had pre-specified three groups of relevant outcomes and further present our findings for each group separately. In particular, psychological and psychosocial outcomes comprise one group, sexual function and satisfaction comprise the second group, and quality of life comprises the last group. This systematic review includes a qualitative but not a quantitative synthesis, because of the expected heterogeneity in the scales used to assess the outcomes of interest. Sensitivity analysis was not performed. Reporting of this systematic review follows the PRISMA guidelines [7].

With regard to risk of bias assessment, non-randomized epidemiological studies are prone to several sources of bias. For the purpose of this systematic review, we pre-specified the following sources of bias as potentially relevant: confounding due to differences between the patients and control group; uncertainty about the patients’ diagnosis; selection bias/missing data due to loss of follow-up data; outcomes measurement; selective reporting. Since MRKH diagnosis cannot be concealed from the study participants, bias due to the non-blinded study design was regarded as present in all of the reviewed studies. Apart from sources of bias, the following were regarded as potential sources of heterogeneity between individual studies’ results: age at patient assessment; surgical or non-surgical treatment that has been applied; time interval since treatment; the country in which the study took place. Data regarding the aforementioned sources of bias and heterogeneity were recorded for all the included studies by two independent reviewers. No overall statement for risk of bias per study was provided because it is unclear whether and how the individual sources of bias should be weighted. As follows, certainty in the body of evidence for each outcome was subjectively assessed via discussion between all authors of this systematic review.

## 3. Results

**Figure 1:**
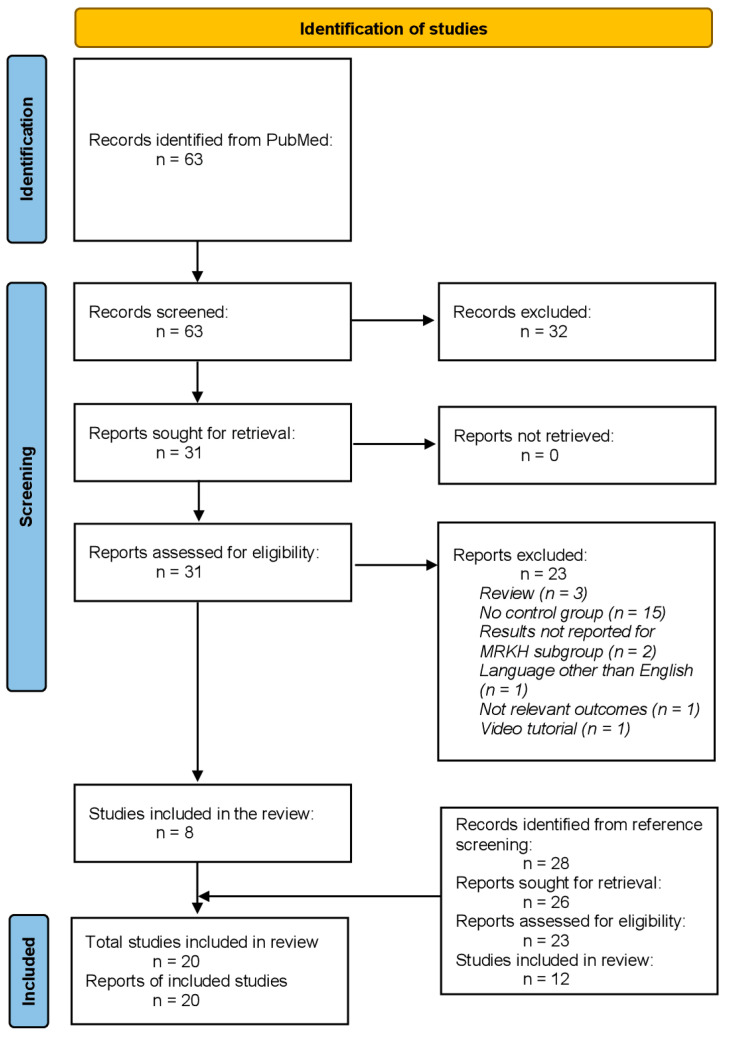
Flow diagram showing number of titles and abstracts identified and screened, and full-text research papers assessed for eligibility and included in the qualitative synthesis.

In total, our search identified 63 records. Of these, 32 were excluded at the screening phase, as they clearly did not meet our inclusion and exclusion criteria based on their title and abstract. At the eligibility phase, 31 full-text articles were assessed. Of these, eight were relevant for our literature review. In addition, after screening the reference list of the included studies and those of the excluded reviews, 28 potentially relevant records were identified. Of these, 26 were assessed in full text and, finally, 12 studies were included in this systematic review. In total, 20 human studies were included in this literature review (Figure 1). Among these 20 studies, 10 studies examined psychological and psychosocial outcomes, 14 examined sexual function and satisfaction in women with MRKH syndrome, and 6 reported on the quality of life of these patients. In 11 out of 21 studies, patients had received surgical treatment for neovagina creation; in 1 study, dilation therapy had been previously applied; in 7 studies, it was unclear which therapeutic intervention had been applied, or different therapeutic interventions had preceded the study; and in 1 study, patients were assessed prior to any treatment. Average age at outcomes assessment varied between 18 and 39.2 years and the time interval since treatment ranged from 6 months to a maximum of 20 years (Table A1).

### 3.1. Psychological and Psychosocial Outcomes

A total of 10 studies including 518 patients have examined the psychological and psychosocial impact of MRKH (Table A1). These studies were published between 1982 and 2021. The psychological and psychosocial impact of MRKH was assessed using questionnaires, including the Patient Health Questionnaire (PHQ), the Scale for the Detection of Self-Acceptance (SESA), and other internationally known, validated tools (Table A2).

In a study by Heller-Boersma et al., in which the Symptom Checklist (SCL-90–R), the Rosenberg Self-Esteem Scale (RSES), the Eating Disorder Inventory (EDI), and the Inventory of Interpersonal Problems (IIP–32) were used, it was found that MRKH patients had significantly higher scores on the first three questionnaires, indicating a higher prevalence of anxiety and depression symptomatology, eating disorders such as bulimia, and a lower self-esteem [8]. However, it should be noted that this study was embedded within a randomized control trial (RCT) that examined the effectiveness of cognitive behavioral group treatment in MRKH patients [8]. Patients that consented to participate in this study perceived a psychological intervention as potentially beneficial for them and were, therefore, more likely to experience anxiety or depression symptoms in comparison with the MRKH patients that did not consent to participate in this study. Thus, the effect of MRKH syndrome on patients’ psychology might be overestimated in the study by Heller-Boersma et al. [8]. Nonetheless, in line with these findings, Chen et al. concluded that MRKH patients were more likely to suffer from depressive symptoms compared with the control group, using the Patient Health Questionnaire-9 (PHQ-9); additionally, Liao et al. demonstrated that the MRKH group had higher mean scores for anxiety, as captured in the Hospital Anxiety and Depression Scale (HADS), compared with the standardization sample [9,10]. Similarly, Fliegner et al. reported that MRKH patients had a higher score in the Social Insecurity Scale and Sexual Insecurity Scale [11]. In the study by Laggari et al., MRKH patients had a greater risk for depressive and anxiety symptoms, but the association was not significant after adjustment for socioeconomic status and chronological age, respectively [12]. On the contrary, other studies have failed to demonstrate any significant psychological or psychosocial impact of MRKH. Rall et al. concluded that there is no evidence for a somatization disorder, no association with depressive or somatic disorders, and a lack of body image disturbance in the MRKH patients group [13]. Similarly, Weijenborg et al., Gatti et al., and Rabboch et al. found no significant difference between the MRKH and the control group, using numerous questionnaires, including the Rosenberg Self-Esteem Scale (RSES) and the Beck Depression Index (BDI) (Table A1) [14,15,16]. Interestingly, Leithner et al. found significantly fewer somatic symptoms and less psychological impairment in the MRKH group compared with the control group and no significant difference regarding body image.

It is evident that the results from studies examining the psychological and psychosocial impact of MRKH syndrome exhibit great heterogeneity [17]. Potential sources of this heterogeneity include the different age at outcomes’ assessment (Table A1); for example, Rall et al. examined patients whose mean age was 19.9, whereas Weijenborg et al. examined patients whose mean age was 39.2 years [13,14]. In addition, the differences in treatment prior to the study as well as the use of different questionnaires may have contributed to the observed heterogeneity.

The results of the reviewed studies may have been influenced by systematic bias, especially due to confounding by differences between the MRKH and the control group and selection bias (Table A3). Even though, in the smaller studies, the vast majority of the invited MRKH patients were enrolled, loss of follow-up data reached 80.3% in other studies (Table A3). Nonetheless, all studies used validated tools to assess the outcomes, and there was none or little uncertainty regarding MRKH diagnosis in the majority of the reviewed studies (Table A3).

Taking all the aforementioned factors into consideration, we are moderately confident that MRKH syndrome probably affects psychologically and/or psychosocially the patients, but further research is needed in order to estimate the extent to which MRKH has a psychological and psychosocial impact on adolescents. 

### 3.2. Sexual Function Outcomes

Fourteen studies evaluated the impact of MRKH syndrome on the sexual function and satisfaction of patients, using a variety of questionnaires and validated tools (Table A1 and Table A2). The studies were published between 1982 and 2021 and included a total of 625 patients. Twelve out of fourteen studies used the Female Sexual Function Index (FSFI). The FSFI evaluates 5 domains: arousal, satisfaction, desire, pain, and lubrication [18]. Other questionnaires that were used include the following: the Female Genital Self-Image Scale (FGSIS), used by Weijenborg et al. and Pastor et al.; the Sexual Arousability Inventory (SAI), used by Raboch et al.; and a structured clinical interview, “Psychosexual biography”, used by Beisert et al. [14,16,19,20]. 

Four studies, those by Raboch, Liu, Zhu, and Cheikhelard, which recruited patients that had been surgically treated almost exclusively, showed no difference in sexual function between the MRKH and the control groups [16,21,22,23]. Interestingly, in the study by Leithner et al., the MRKH group had marginally higher FSFI scores postoperatively [17]. On the contrary, in three studies (Rall et al., Fliegner et al., Liao et al.), MRKH patients had lower mean total FSFI scores and lower scores in all of the subscales compared with the reference sample, indicating reduced sexual function and stronger limitations within the domains of desire, arousal, lubrication, orgasm, and pain [10,11,13]. In two of these studies (Rall et al. and Fliegner et al.), the majority of patients had been previously operated upon, whereas in the study of Liao et al., previous treatment of the participants was unclear [10,11,13]. An important finding of Rall was that the FSFI total scores in MRKH patients remained lower even after postoperative improvement [13]. In addition, two studies by Nadarajah and Fedele, respectively, associated MRKH with an inability to maintain lubrication and to experience orgasm, as well as with stronger pain during sexual intercourse [24,25]. The other domains of FSFI showed no significant difference [24,25]. Notably, Fedele et al. recruited MRKH patients postoperatively, whereas Nadarajah et al. recruited patients after treatment with dilators [24,25]. Csermely et al. reported that the MRKH group postoperatively showed significantly less lubrication and had significantly more discomfort (pain) during sexual intercourse, but had no other significant difference compared to the control group [26]. In line with the aforementioned findings, Weijenborg and Pastor studied MRKH patients postoperatively and associated MRKH syndrome with higher incidence of Female Sexual Dysfunction (FSD), the presence of some or higher levels of pain during intercourse, and significantly lower self-esteem, as the assessment of genital perception, using the Female Genital Self-Image Scale (FGSIS), showed a significantly lower score [14,19]. Lastly, Beisert et al. noted that the frequency of vaginal and oral intercourse as well as the frequency of orgasms during vaginal intercourse was significantly lower in the MRKH group [20]. It is worth mentioning that, in the same study, women with MRKH were found to engage in vaginal and oral intercourse later compared with the controls. All other aspects of the clinical interview showed no significant difference between the two groups [20]. 

With regard to sexual functioning outcomes among women with MRKH, some heterogeneity is observed, since four out of fourteen studies showed no significant difference between the two groups. An important source of this heterogeneity is the difference in type of treatment and age at which the MRKH patients had been treated (Table A1). For example, in the study by Nadarajah et al., 2005 patients had been treated with vaginal dilatators at mean age 20.5 years; in the study by Fedele et al., 2007, patients had been surgically treated with modified laparoscopic Vecchietti operation at a mean age of 17 years; and in the study by Liu et al., patients had received laparoscopic Davydov vaginoplasty at a mean age of 24 years [21,24,25]. It is evident that this difference in treatment and age at treatment could affect the sexual functioning outcome and the way that the MRKH population perceives and experiences sexual intercourse. An additional source of heterogeneity among the reviewed studies is the use of different questionnaires to assess the targeted outcomes. 

Regarding bias assessment, all the reviewed studies used validated questionnaires to examine sexual function outcomes (Table A3). Concerning selection bias, 0–75.5% of the invited MRKH patients did not finally participate (Table A3). Furthermore, in 5 out of 14 studies, the diagnostic algorithm for MRKH syndrome was not explicitly stated (Table A3). Last but not least, confounding factors—attributable to sociodemographic differences between the MRKH and control group—likely affected the results of the reviewed studies, even though 9 out of 14 studies enrolled age-matched controls (Table A3).

We are, therefore, moderately confident that reviewed evidence supports an association between MRKH syndrome and poorer sexual function outcomes, even though this was not the case in all the conducted studies. 

### 3.3. Quality of Life (QoL) Outcomes

Six studies have reported on the quality of MRKH patients’ life (Table A1). These studies included data from 312 patients in total and were published between 2002 and 2021. Leithner et al., Cheikhelard et al., and Kimberley et al. used the World Health Organization’s Quality of Life Questionnaire (WHOQOL-Bref); Rall et al. and Liao et al. used the short version of the SF-36 Health Survey (SF-12) and the Mental Health Component Summary Score (MCS); and Kaloo used the Sintonen 15D questionnaire of general wellbeing and sexual health (Table A2) [10,13,17,23,27,28]. 

Four out of six studies reported significant impairment of mental-health-related quality of life, and generally indicated poorer mental health of MRKH patients compared with the control groups [10,13,17,23]. The last two studies, by Kaloo and Kimberley, demonstrated no association between MRKH and deterioration of the quality of life [27,28]. Kaloo et al., however, reported that the sexual activity of MRKH women was significantly lower than the average in the normal female Finnish population [28]. It is worth mentioning that, in the studies by Liao, Leithner, Cheikhelard, and Rall, the physical health of MRKH patients did not differ from controls [10,13,17,23]. 

Regarding the physical health component of the quality of life of MRKH patients, the studies report no heterogeneity, since none of the studies found any significant association between MRKH syndrome and a poorer physical health. There is, however, some heterogeneity regarding the mental health component of quality of life and general wellbeing. Differences in power among the studies may explain to an extent this heterogeneity (Table A1). For example, Kimberley studied only 28 MRKH patients and found no association, whereas Cheikhelard studied 131 and reported poorer mental-health-related quality of life; notably, the same questionnaire was used in both studies [23,27]. Furthermore, the use of different tools and questionnaires could have also affected heterogeneity, since the Sintonen 15D questionnaire was used only by Kallo et al., who reported no impact of MRK syndrome on quality of life [28]. 

With regard to risk of bias assessment, issues arise again due to significant differences between the MRKH and the control group; notably, in three out of six studies, general population samples served as control groups (Table A3). In addition, 16.7–67% of eligible MRKH patients were not included in the reviewed studies, indicating that selection bias may have influenced the results (Table A3).

Therefore, we are moderately confident that the reviewed evidence supports that MRKH is associated with a poorer mental-health-related quality of life, but physical-health-related quality of life is not affected by the syndrome. 

## 4. Discussion

In this systematic review of the literature, the psychology, quality of life, and sexual life of patients with MRKH syndrome was studied in comparison to non-affected individuals. Overall, we concluded that MRKH could be associated with a higher prevalence of anxiety and depression symptoms, as well as with social insecurity, in comparison with women of similar age without the condition. Regarding the sexuality of MRKH patients, most studies agreed that MRKH is associated with greater pain and discomfort during sexual intercourse as well as with strong limitations in arousal, lubrication, and orgasm. Moreover, MRKH women with a neovagina experience sexuality-related distress, suffer from sexual dysfunction more often, and report lower sexual esteem and genital self-image compared with controls, reflecting that MRKH women might feel insecure about themselves. Lastly, MRKH patients more commonly experience significant impairment of the mental-health-related quality of life, but physical-health-related quality of life does not seem to be influenced by MRKH.

The impact of MRKH syndrome on psychology, sexual life, and quality of life of patients has been long suspected. The American College of Obstetricians and Gynecologists (ACOG) recommends that all patients with MRKH should be offered psychological counseling and be encouraged to connect with support groups in order to deal with this great emotional burden, and underlines that the psychological impact of the diagnosis should not be underestimated [29]. With regard to psychological counseling, cognitive–behavioral group treatment has been shown to significantly reduce psychological symptoms, as captured in the Symptom Checklist-90 (SCL-90) [30]. Women with the syndrome have agenesis of the uterus and the upper third of their vagina; thus, the main focus of clinical management has traditionally been to increase the vaginal length, either by surgical or by nonsurgical techniques [4]. The aforementioned anatomical abnormalities associated with MRKH can lead to difficulties during sexual intercourse and infertility, causing confusion of identity and negative self-beliefs. The majority of cases are diagnosed during adolescence, when body image and sexual identity are shaped. Permanent loss of bodily integrity, even fertility, and the need for the artificial creation of a neovagina can have an impact on identity and self-evaluation in this sensitive age. Taking into account the emotional distress associated with infertility alone, we can expect that the diagnosis of MRKH—as well as the distress caused by a potential surgical therapy—may compromise emotional wellbeing, relationship outcomes, and sexual function [9]. While living with this condition, depression, anxiety, and even suicidal thoughts can appear [31,32]. The results of this systematic review also support the hypothesis that deep dyspareunia may be associated with the creation of the artificial neovagina.

However, as interesting as the findings of this systematic review may be, several aspects of MRKH impact on psychology, sexual life, and quality of life remain understudied. Most importantly, there is a marked lack of evidence for the period of adolescence, when the diagnosis is usually made. The average age at which assessment was made was less than 22 years old only in four out of the twenty reviewed studies. Furthermore, the vast majority of studies did not include pretreatment data, which is usually the case for adolescent patients, but rather evaluated the patients and assessed the targeted outcomes only after treatment for MRKH syndrome. In addition, many geographical regions and cultures are under-represented in the reviewed studies; in particular, fifteen studies were conducted in Europe, three studies in China, and two in Australia, while patients from Bangladesh were recruited in just one study. Therefore, the majority of patients included in the reviewed studies shared a similar cultural background and similar sexual experiences and perceptions regarding sexual satisfaction and outcomes. It is, therefore, unclear how MRKH syndrome affects psychology, sexual life, and quality of life of patients of non-Western and non-Chinese origin. Furthermore, the extent to which infertility mediates the effect of MRKH syndrome on patients’ psychology has been markedly understudied. Infertility is regarded as one of the most difficult aspects of MRKH syndrome for patients to accept, but uterine transplantation emerges as a therapeutic solution for infertility. Finally, all of the reviewed studies have used psychometric instruments of symptomatic evaluation to assess psychological outcomes, rather than diagnostic instruments. Therefore, MRKH syndrome could be associated with anxiety and depression symptoms, but it remains unknown whether clinical depression and anxiety disorders are more frequent among MRKH patients in comparison with the general population.

To our knowledge, this is the first study that has systematically reviewed quality of life, psychological, and psychosocial outcomes, as well as sexual function outcomes, in MRKH patients in comparison with healthy controls. These outcomes are known to be complex, heterogeneous, and multifactorial in origin. The adaption of a broad search and reference screening strategy is a strength of this literature review, even though the search algorithm in PubMed has not been optimized based on the literature found via the references screening. Other limitations of this systematic review are that the protocol was not registered a priori, and databases other than PubMed, as well as the grey literature, were not searched. In addition, a language criterion was applied, resulting in the exclusion of one potentially relevant paper. Finally, the retrieval of identified research records was incomplete, as three potentially relevant records (all identified from reference screening) were not accessible in full text.

## 5. Conclusions

In conclusion, the results of this systematic review emphasize the need for psychological support and counseling regarding sexual life among MRKH patients. Gynecologists, pediatricians, and other specialists who treat MRKH syndrome are encouraged to take into consideration the challenges of living with MRKH syndrome and to investigate the multiple clinical needs of this population more thoroughly. Depressive symptoms and mood disorder screening in this vulnerable group is of the utmost importance.

## Figures and Tables

**Table 1:** The search algorithm in PubMed.

(“müllerian agenesis” [tiab] OR “mullerian agenesis” [tiab] OR “müllerian aplasia” [tiab] OR “mullerian aplasia” [tiab] OR “müllerian dysgenesis” [tiab] OR “mullerian dysgenesis” [tiab] OR (“Mayer” [tiab] AND “Rokitansky” [tiab]) OR “MRKH” [tiab] OR “vaginal agenesis” [tiab] OR “vaginal aplasia” [tiab] OR “uterine aplasia” [tiab] OR “MURCS” [tiab] OR “rokitans*” [tiab])
AND
(“sexual” [tiab] OR “psychological” [tiab] OR “psychosocial” [tiab] OR “emotional” [tiab] OR “depress*” [tiab] OR “anxiety” [tiab] OR “quality of life” [tiab] OR “quality-of-life” [tiab] OR “QoL” [tiab] OR “well-being” [tiab] OR “gender” [tiab])
AND
(hasabstract[text] AND “loattrfull text” [sb])
NOT
(animals [mh] NOT humans [mh])

Asterisk (*) in PubMed is the truncation symbol and represents any group of characters, including no character; it is used in search algorithms, in order to search for all terms that begin with that basic word root.

## Data Availability

Not applicable.

## Appendix A

**Table A1:** Summary of findings of the reviewed studies.

Original Study	Country	Assessment Year	Age at Outcomes’ Assessement	Treatment	Time Period Between Treatment and Assessment	Sample Size	Recruitment Strategy	Summary of Findings for Psychological and Psychosocial Outcomes	Summary of Findings for Sexual Function Outcomes	Summary of Findings for Quality of Life (QoL) Outcomes
Rall et al., 2021 [13]	Germany	September 2009–December 2015	mean 19.9 years (SD 5.3)	Laparoscopically assisted creation of a neovagina (modified Vecchietti technique)	3 timepoints: before surgery, follow-up visits at 6 months and 12 months after surgery	82 MRKH patients compared to several reference samples	Consecutive patients invited	(1) the German version of the Patient Health Questionnaire (PHQ): Compared to the healthy reference sample there is no evidence for a somatization disorder in the MRKHS patients. On the other hand, the MRKHS patients differ significantly from the reference sample with mental disorders indicating a lack of a depressive or somatic disorder. (2) the questionnaire concerning body image (FKB-20): MRKHS patients are significantly below the medical students reference and over the mean of the patient reference sample, indicating lack of body image disturbance. (3) the Scale for the detection of self-acceptance (SESA): Significant indicators for a worse self-esteem of the MRKHS patients compared to the healthy reference sample are found pre-operatively but not post-operatively. The comparison with the depressive reference sample shows better values for the MRKHS patients for all three timepoints	the German version of the Female Sexual Function Index (FSFI): Lower FSFI-Total Score in MRKH patients, which improved post-operativelly. The MRKHS patients show stronger limitations within the domains desire, arousal, lubrication, orgasm, and pain compared to the reference sample.	the short version of the SF-36 Health Survey (SF-12): Physical Component Summary Score (PCS): MRKHS patients do not differ from the reference sample. Mental Health Component Summary Score (MCS): the MRKHS patients had lower scores, indicating significant impairment of the health-related mental quality of life.
Chen et al., 2020 [9]	China	unclear, but not earlier than 2018	MRKH patients: mean 25.8 years (SD 4.6) Controls: mean 26 years (SD 5.1)	33 (23.4%) patients no treatment 67 (47.5%) patients nonsurgical dilation 41 (29.1%) patients vaginoplasty surgery	unclear	141 MRKH patients vs. 178 age matched controls	Consecutive patients invited	Depressive Symptoms assessed via Patient Health Questionnaire-9 (PHQ-9): The PHQ-9 score (median and IQRs) was 7.0 (4.5–11.0) in MRKH patient group while 6.0 (3.0–9.0) in the agematched control group, the former being significantly higher than the latter (*p* = 0.015). Altogether, 75.2% (106/141) of patient group and 61.2% (109/178) of control group suffered from depressive symptoms (PHQ-9 score ≥ 5).	-	-
Weijenborg et al., 2019 [14]	the Netherlands	November 2015–May 2017	MRKH: mean 39.2 years (SD 13.8) Controls: mean 36.7 years (SD 11.1)	Surgical	2 MEKH ≤ 1 years, 11 MRKH 1–5years, 6 MRKH 5–10 years, 32 MRKH > 10 years, Missing 3)	54 MRKH patients vs. 79 age matched controls	Via the Dutch peer support group ‘The foundation of MRK women’ and patients from the departments of Gynecology in three University Medical Centers in the Netherlands	(1) Symptom Checklist-90 (SCL-90): no significant difference (2) Hospital Anxiety and Depression Scale (HADS): no significant difference (3) Rosenberg’s SelfEsteem Scale (RSES): no significant difference (4) Relational functioning: The subscale relationship satisfaction of the Maudsley Marital Questionnaire (MMQ): no significant difference	(1) Female Sexual Function Index (FSFI): no significant difference in total score. MRKH reported lower score on subscale pain (->more pain) (*p* < 0.05) (2) Female Sexual Distress Scale (FSDS): no significant difference (3) Female Sexual Dysfunction (FSD) is diagnosed when FSFI score <26.55 and FSDS score >15: significantly more MRKH women than controls suffered a FSD (*p* < 0.05, OR: 2.654, 95% CI: 1.088–6.471) (4) Genital Pain Rating (GPR): More MRKH women than controls reported the presence of some or higher levels of pain during intercourse as assessed with the GPR (MRKH women: N = 31, 65% versus controls: N = 30, 41%; *p* < 0.05, OR: 2.675, 95% CI: 1.261–5.672) (5) Sexual Esteem subscale of the Multidimensional Sexuality Questionnaire (MSQ): MRKH women scored significantly lower on the sexual esteem subscale (*p* < 0.01) (6) Female Genital Self-Image Scale (FGSIS): MRKH women scored significantly lower on the FGSIS (*p* < 0.01)	-
Cheikhelard et al., 2018 [23]	France	October 12–April 2015	mean 26.5 years (SD 5.5)	Surgery (N = 84) Dilation therapy (N = 26) Intercourse (N = 20)	minimum one year -maximum 20 years	131 MRKH patients vs. French general population	Physicians experienced in MRKH syndrome from 16 centers invited their patients	-	FSFI: Global FSFI scores in MRKH syndrome patients were not different to the general population.	WHOQoL-Bref: The global WHOQOL-BREF score was not different. Satisfaction toward health score was significantly lower in MRKH. Regarding dimension, physical heatlh did not differ, but the psychological and the social dimensions scores were lower in MRKH.
Pastor et al., 2016 [19]	Czech Republic	2015	mean 25.8 years (SD 4.3, range 17–38)	Laparoscopic Vecchietti vaginoplasty	operation 2004–2013	42 MRKH patients vs. 45 age-matched, sexually active, childless patients with intrauterine delivery system containing levonorgestrel	Consecutive patients invited	-	(1) Psychosexual function-> Female Sexual Distress Scale Revised (FSDS-R), Female Sexual Function Index (FSFI), and a semistructured interview: The FSDS-R score was significantly higher in operated women compared with the control group (14.5 ± 6.5 vs. 6.5 ± 4.6). Sexual function as indicated by FSFI total score was similar between groups, which did not significantly differ statistically (29.9 ± 2.7 vs. 30.0 ± 2.1). However, the groups differed in four (desire, lubrication, orgasm, and comfort) of the six FSFI domains, with women with a neovagina reporting significantly more frequent orgasms and higher sexual desire but less lubrication and more discomfort (pain) during intercourse. (2) Genital self-image -> Female Genital Self-Image Scale (FGSIS): FGSIS assessment of genital perception showed a significantly lower score in women with a neovagina (22.0 ± 2.4 vs. 23.5 ± 2.3). (3) frequency of vaginal intercourse, no statistically significant difference (4) frequency of masturbation, no statistically significant difference (5) frequency of orgasm, no statistically significant difference (6) Satisfaction from sexual life, no statistically significant difference	-
Leithner et al., 2015 [17]	Austria	September 2009–February 2010	unclear	Neovaginoplasty according to Wharton-Sheares-George at a mean age of 22.9 years (SD 5.7)	3–77 months	10 MRKH patients vs. 20 age- and education-matched female controls	Consecutive patients invited	(1) Patient Health Questionnaire (PHQ): MRKH showed significantly less somatic symptoms than controls. Depressive symptomatology and psychosocial burden were not significantly different between groups. (2) Brief Symptom Inventory (BSI): MRKH had significantly lower “Positive Symptom Total” scores indicating less psychological impairment than controls. Severity of symptoms and basic psychological impairment were not different between groups. (3) body image -> Fragebogen zur Beurteilung des eigenen Körpers (FBeK):)In none of the four scales (“Attractiveness/Self-Confidence”; “Accentuation of Physical Appearance”; “Insecurity/Concern”, “Physical/Sexual discomfort”) a significant difference was observed.	FSFI: Marginally higher FSFI scores in MRKH	WHOQoL-Bref: No group differences were found on the domains “Physical health”, “Social relationships”, “Environment”, and “Global”. Within the domain “Psychological”, MRKHS scored significantly higher than controls.
Beisert et al., 2015 [20]	Poland	unclear	MRKH patients: mean 22.7 years Controls: mean 24.2 years	21 out of 31 surgical treatment at a mean age of 20.8 (range 17.9–26.1)	unclear	31 MRKH patients vs. 31 controls matched for age, education, and place of living	Unclear	-	Structured Clinical Interview "Psychosexual biorgaphy" by M. Beisert that examines: (1) frequency of masturbation, no difference, *p* = 0.653 (2) frequency of petting, no difference, *p* = 0.473 (3) frequency of vaginal intercourse, MRKH less frequently, *p* < 0.001 (4) frequency of oral intercourse, MRKH less frequently, *p*-0.016 (5)frequency of anal intercourse, no difference, *p* = 0.384 (6) age at 1st masturbation, no difference, *p* = 0.053 (7) age at 1st petting, MRKH later, *p* = 0.003 (8) age at 1st vaginal intercourse, MRKH later, *p* < 0.001 (9) age at 1st oral intercourse, MRKH later, *p* = 0.023 (10) age at 1st anal intercourse, no difference, *p* = 0.842 (11) frequency of orgasms during petting, no difference, *p* = 0.051 (12) frequency of orgasms during vaginal intercourse, MRKH less frequently, *p* = 0.034 (13) frequency of orgasms during oral intercourse, no difference, *p* = 0.066 (14) frequency of orgasms during anal intercourse, no difference, *p* = 0.219 (15) frequency of autoerotic activity in adolescence, no difference, *p* = 0.661 (16) frequency of autoerotic activity in adulthood, no difference, *p* = 0.711 (17) level of sexual arousal in adolescence, no difference (exact *p* not reported	-
Fliegner et al., 2013 [11]	Germany	March 2010–July 2011	median 22 years (interquartile range 19–27)	46 patients had surgery for neovagina (several techniques) 3 patients report no prior surgery	unclear	49 MRKH patients vs. 145 controls	Mixed methods used (via support groups, professionals in the field of gynecology and endocrinology, Internet announcement, Tübingen Women’s Health Clinic)	(1) Feelings of Inadequacy in Social and Sexual Situations -> FUSS (Social insecurity Scale and Sexual Insecurity Scale): FUSS-Social -> No difference, FUSS-sexual -> higher score in MRKH patients (2) Rosenberg’s Self Esteem Scale (RSES): higher RSES score in MRKH patients	FSFI: Lower FSFI scores in MRKH patients	-
Zhu et al., 2013 [22]	China	from June 2007 to January 2013	mean 24.6 years (SD 2.4)	Vaginoplasty with tissue-engineered biological material (acellular dermal matrix) from June 2006 to January 2012 (mean age 23.6, SD 2.4 years)	12 months	24 MRKH patients vs. 24 age matched women	Consecutive patients invited	-	FSFI: The mean total score for MRKH was 26.7 (SD 3.5) vs. 25.6 (SD 7.4) for controls. All mean domain scores were similar (desire, arousal, lubrication, orgasm, and global sexual satisfaction; *p* > 0.1 for all).	-
Liao et al., 2011 [10]	UK	unclear	median 21.7 years (range 18–52)	Unclear	unclear	56 MRKH patients (36 for FSFI and MSQ) vs. standarization sample	Consecutive patients invited	Anxiety and Depression ->HADS: higher mean scores for anxiety compared with the standardization sample (8.4 vs. 4.3). No significant difference for depression (4.0 vs. 4.4, *p* = 0.64).	(1) FSFI: The mean total score was lower than the standardization population, indicating reduced sexual function (23.4 vs. 30.5). Lower scores in all of the subscales equally: desire, arousal, lubrication, orgasm, satisfaction, and pain. (2) psychological tendencies associated with sexual relationships -> MSQ: Of the 12 subscales, scores were lower than reference for sexual esteem (50%) and sexual preoccupation (53%), and greater than reference for sexual depression (205%), sexual anxiety (172%), and fear of sexual relations (146%). Scores for sexual monitoring, internal and external sexual control, sexual consciousness, motivation, satisfaction, and assertiveness were within 30% of the reference value.	SF-12: physical health (PCS-12): higher mean scores than the standardization sample (55.8 vs. 50.9). mental health (MCS-12):lower mean scores (poorer mental health) compared with the standardization sample (42.0 vs. 52.1).
Csermely et al., 2011 [26]	Hungary	unclear	unclear	Modified laparoscopic Vecchietti operation from 1998–2007 at the age of 16–26	2–11 years	23 MRKH patients vs. 25 age matched controls	Consecutive patients invited	-	FSFI: The total FSFI scores did not differ from that of the control group. In four (desire, arousal, orgasm, and satisfaction) of the six domains the operated patients reported on similar results as controls. MRKH patients with neovagina showed significantly less lubrication and had significantly more discomfort (pain) during sexual intercourse.	-
Kimberley et al., 2010 [27]	Australia	unclear	median 23 years (range 16–71)	Either surgical or dilators	unclear (max 20 years)	28 MRKH patients vs. Australian population average	Consecutive patients invited	-	-	WHOQoL-Bref: similar results
Gatti et al., 2010 [15]	Italy and Bangladesh	unclear	Bangladesh: MRKH mean 27.8 years (SD 1.3) and controls mean 27.8 years (SD 1.4) Italy: MRKH mean 25.2 years (SD 1.2) and controls mean 25.2 years (SD 1.1)	Total sigmoid vaginal replacement between 1995 and 2008 at a mean age of 18.4 years (range 10–29)	unclear	Bangladesh: 37 MRKH patients vs. 20 controls Italy: 6 MRKH patients vs. 10 controls Controls: women who underwent surgery for minor pathological conditions	Consecutive patients invited	(1) Rosenberg Self-Esteem Scale (RSES), no difference (exact *p* not reported) (2) Beck Depression Index (BDI), no difference (exact *p* not reported) (3) Cohen Test for Life Managemenet Ability (CTLMA), no difference (exact *p* not reported)	-	-
Heller-Boersma et al., 2009 [8]	UK	unclear	MRKH: mean years 27.9 (SD 1.0) Controls: mean 27.8 years (SD 1.5)	Unclear	unclear	66 MRKH patients vs. 31 controls recruited from a London City International Church congregation and from the City University (London) student population.	MRKH register at the U.K. National Centre for Adolescent and Adult Women with Congenital Abnormalities of the Genital Tract were invited in a randomized, controlled trial (RCT) of group cognitive-behavioral therapy	(1) The Symptom Checklist (SCL-90–R): MRKH women had significantly higher scores on the subscales Phobic Anxiety and Psychoticism (interpersonal alienation), with a similar trend for the subscales Depression (*p* = 0.089) and Anxiety (*p* = 0.087). (2) The Rosenberg SelfEsteem Scale (RSES): MRKH women had significantly higher scores (i.e., lower selfesteem) (*p* = 0.007). (3) The Inventory of Interpersonal Problems (IIP–32): no difference between groups (4) The Eating Disorder Inventory (EDI): MRKH women had significantly higher EDI total scores (*p* = 0.018), and higher scores on the subscales Interoceptive Awareness (*p* = 0.001), Interpersonal Distrust (*p* = 0.027), Ineffectiveness (*p* = 0.001), and Bulimia (*p* = 0.017).	-	-
Laggari et al., 2009 [12]	Greece	unclear	MRKH: mean 18 years (SD 1.4) Controls: mean 17 years (SD 2.2)	No prior treatment	does not apply	5 MRKH patients vs. 22 controls	Unclear	(1) The Beck Depression inventory (BDI): MRKHS higher scores (*p* < 0.05). After adjustement for stressfull life events OR= 1.12 to have higher scores on STAI-Gr than the control group. After adjustment for chronological age (OR = 1.11). The association was not significant after adjustment for socio-economic status. (2) The State-Trait Anxiety Inventory (STAI): MRKHS higher scores on BDI after adjustment for stressful life events (OR = 1.19) and socio-economic status (OR = 1.40, *p* < 0.05), but this significance disappeared after adjustment for chronological age.	-	-
Liu et al., 2009 [21]	China	unclear, but not earlier than 2005	MRKH: mean 24.6 years (SD 3.8, median 24.5, range 19–35) Controls: mean 26.8 years (SD 4.1, median 26, range 18–37)	Laparoscopic Davydov vaginoplasty from January 2005 to February 2008 at a mean age of 24 years (range 19–35)	median 25 months (range 4–40 months)	24 MRKH patients vs. 50 randomly selected, age matched healthy women	Consecutive patients invited	-	FSFI: No statistically significant difference in total score or the six domains, even though the pain score was marginally lower in the cases.	-
Fedele et al., 2007 [4]	Italy	2000–2005	mean 21.5 years (SD 1.8)	Laparoscopic Vecchietti modified operation from June 1993 to December 2004 at a mean age of 17 years	12 months	27 MRKH patients vs. 27 age matched controls	Consecutive patients invited	-	FSFI: Significantly lower total SFSI score for MRKH patients (29, SD 3.2 vs. 31, SD 2.4). Significantly lower score for the domains on lubrification (5, SD 0.9 vs. 5.6, SD 0.5), orgasm (4.6, SD 1 vs. 5, SD 0.6), and comfort (5, SD 0.9 vs. 5.5, SD 0.6). No significant difference was found between the patients and the controls in the domains regarding desire, arousal, and satisfaction.	-
Nadarajah et al., 2005 [24]	UK	unclear	mean 26.6 years (range 17 to 46)	Vaginal dilatators at mean age 20.5 years (range 16–44)	mean 5.4 years	60 MRKH patients vs. 129–131 controls (no details for them)	Consecutive patients invited	-	Female Sexual Function Index (FSFI): No significant difference for the domains sexual desire, sexual arousal and satisfaction with a sexual relationship. There was a significant difference for vaginal lubrication with the study population reporting reduced frequency as well as greater difficulty in becoming lubricated. The most significant difference in this domain was in their inability to maintain lubrication until completion of sexual intercourse (*p* < 0.001). There was a significant difference in the Rokitansky patients’ ability to reach orgasm.Among all the domains, the most significant difference was seen with regards to pain during and following vaginal penetration (*p* < 0.001).	-
Kaloo et al., 2002 [28]	Australia	unclear	unclear	Laparoscopic Vecchietti vaginoplasty	up to 3 years	5 MRKH patients vs. normal female Finnish population	Consecutive patients invited	-	-	The Sintonen 15D questionnaire of general well being and sexual health revealed an average score 0.969 (SD = 0.029), not statistically different from normal female Finnish population aged 19 to 39 years (0.960 (SD = 0.047)). However, at the sexual activity dimension the average score was 0.769 (SD = 0.130), significantly poorer than the average of 0.968 (SD = 0.102) in the normal female Finnish population.
Raboch et al., 1982 [16]	Czechoslovakia	unclear	MRKH: mean 23.3 years (range 20–28) Controls: mean 24.9 years (range 28–30)	Surgical	mean 3.3 years (range 0.25–9)	12 MRKH patients vs. 22 age matched controls	Probably consecutive patients invited	Questionnaire N5 (Engelsman, 1966), which investigates the occurrence of neurotic symptoms such as sleep disturbances, fatigue, sweatiness, irritability, and heart palpitations: no significant difference	(1) HTDW (Heterosexual Development of Women; Mellan, 1980), higher scores representing an acceleration and lower scores a retardation of sexual development: no significant difference (2) SFW (Sexual Function of Women, Mellan, 1978b): no significant difference (3) SAI (Sexual Arousability Inventory, Hoon et al., 1976, modified by Mellan, 1978a, b): no significant difference	-

**Table A2:** Scales and questionnaires used per outcome domain.

	Scales Used
Psychological and Psychosocial Outcomes	Patient Health Questionnaire (PHQ): Assesses depressive symptoms Patient Health Questionnaire-9 (PHQ-9): Assesses depressive symptoms Body Image Questionnaire-20 (FKB-20): Assesses the body image perception Self-Acceptance Scale (SESA): Assesses self-acceptance Symptom Checklist-90 (SCL-90): Assesses a broad range of psychological problems Hospital Anxiety and Depression Scale (HADS): Assesses anxiety and depressive symptoms Rosenberg’s Self Esteem Scale (RSES): Assesses self-esteem Maudsley Marital Questionnaire (MMQ): Assesses the relational functioning (the subscale relationship satisfaction has been used) Brief Symptom Inventory (BSI): Assesses a broad range of psychological problems (9 primary symptom dimensions: somatization, obsession-compulsion, interpersonal Sensitivity, depression, anxiety, hostility, phobic anxiety, paranoid ideation, psychoticism) Fragebogen zur Beurteilung des eigenen Körpers (FBeK): Assesses body image Social insecurity Scale and Sexual Insecurity Scale (FUSS): Assesses feelings of inadequacy in social and sexual situations Beck Depression Index (BDI): Assesses depressive symptoms Cohen Test for Life Managemenet Ability (CTLMA): Assesses role limitation due to physical and emotional problems, pain and general health perception The Inventory of Interpersonal Problems (IIP–32): Assesses interpersonal sifficulties The Eating Disorder Inventory (EDI): Assesses symptoms relared to eating disorders The State-Trait Anxiety Inventory (STAI): Assesses trait and state anxiety Questionnaire N5 (Engelsman, 1966): Assesses the occurrence of neurotic symptoms such as sleep disturbances, fatigue, sweatiness, irritability, and heart palpitations
Sexual Function Outcomes	Female Sexual Function Index (FSFI) Female Sexual Distress Scale (FSDS) Female Sexual Distress Scale Revised (FSDS-R) Female Sexual Dysfunction (FSD) Female Genital Self-Image Scale (FGSIS) Genital Pain Rating (GPR) Multidimensional Sexuality Questionnaire (MSQ): Assesses psychological tendencies associated with sexual relationships (12 subscales: sexual esteem, sexual preoccupation, internal sexual control, sexual consciousness, sexual motivation, sexual anxiety, sexual assertiveness, sexual depression, external sexual control, sexual monitoring, fear of sexual relationships, and sexual satisfaction) Semi-structured interview Frequency of vaginal intercourse Frequency of masturbation Frequency of orgasm Satisfaction from sexual life Structured Clinical Interview “Psychosexual biography” by M. Beisert that examines: frequency of masturbation, frequency of petting, frequency of vaginal intercourse, frequency of oral intercourse, frequency of anal intercourse, age at first masturbation, age at first petting, age at first vaginal intercourse, age at first oral intercourse, age at first anal intercourse, frequency of orgasms during petting, frequency of orgasms during vaginal intercourse, frequency of orgasms during oral intercourse, frequency of orgasms during anal intercourse, frequency of autoerotic activity in adolescence, frequency of autoerotic activity in adulthood, level of sexual arousal in adolescence Heterosexual Development of Women (HTDW); Mellan, 1980 Sexual Function of Women (SFW); Mellan, 1978 Sexual Arousability Inventory (SAI); Hoon et al., 1976, modified by Mellan, 1978
Quality of Life (QoL) Outcomes	The Short Form (12) Health Survey (SF-12) WHOQoL-Bref The 15D questionnaire

**Table A3:** Risk of bias assessment among the reviewed studies.

Original Study	Confounding Due to Differences between the Patients and Control Group	Uncertainty about the Patients’ Diagnosis	Missing Data Due to Loss of Follow-Up	Outcomes’ Measurement	Selective Reporting
Rall et al., 2021 [13]	PHQ-D: (1) Continuous Scales: 2 control groups (1st: 357 healthy participants, 2nd: 117 patients with mental disorders). Males included in both control groups, mean age of control groups significantly higher (41.9 in controls vs. 19.9 in MRKH) (2) Categorical Scales: Control group’s sample size unknown, only females, significantly higher range of age (18–34) compared to MRKH patients (19–21) FKB-20: 2 control groups (1st: 56 female medical students, 2nd: 253 female patients). 2nd control group of older age; probably also the 1st control group FSFI-d: 129–131 controls of older age (mean 39.7 years vs. 19.9 in MRKH patients) SF-12: 4 control groups (2 of healthy participants; n1 = 123 of age 14–20, and n2 = 473 of age 21–30, and 2 with chronic diseases, n3 = 46 of age 14–20 and n4 = 227 of age 21–30). Males included in all 4 control groups. SESA: 2 control groups (1st-> 311 healthy controls both males and females of median age 29 years, 2nd-> 45 depressive controls, both males and females of median age 45 years)	None (diagnosis confirmed during laparoscopically assisted creation of a neovagina)	82 patients included out of 160 invited (48.8% loss of follow-up)	Only validated tools (PHQ-D, FKB-20, FSFI-d, SF-12, SESA)	Not suspected
Chen et al., 2020 [9]	Age-matched controls	Uncertainty present (diagnostic algorithm not reported)	141 patients included out of 218 invited (35.3% loss of follow-up)	Only validated tools (PHQ-9)	Not suspected
Weijenborg et al., 2019 [14]	Age-matched healthy women without the condition were enrolled. However, all participants had to be at least 18-years old and had to live in a steady heterosexual relationship, whereas the same inclusion criterion is not explicitely reported for the control group.	Little uncertainty present (All women with MRKH syndrome who were known by the Dutch peer support group “The foundation of MRK women”)	Unclear (number of invited MRKH patients is not reported)	Only validated tools (FSFI, FSDS, GPR, SES, a subscale of the MSQ, FGSIS, SCL-90, HADS, RSES, the subscale relationship satisfaction of the MMQ)	Not suspected
Cheikhelard et al., 2018 [23]	The general French population as control group (sociodemographic characteristics may differ)	None	131 patientas included out of 397 invited (67% loss of follow)	Only validated tools (FSFI, WHOQoL-Bref)	Not suspected
Pastor et al., 2016 [19]	Age-matched, childless controls. However, controls were using intrauterine delivery system containing levonorgestrel 13.5 mg, and had higher total number of sexual partners	None (diagnosis confirmed during laparoscopic Vecchietti vaginoplasty)	42 patients included out of 95 invited (56.7% loss of follow-up)	Validated tools (FSDS-R, FSFI, FGSIS) and unvalidated tools (structured interview)	Not suspected
Leithner et al., 2015 [17]	Age- and education-matched female control subjects	None (diagnosis confirmed during neovaginoplasty according to Wharton-Sheares-George)	10 patients included out of 17 invited (41.2% loss of follow-up)	Only Validated tools (FSFI, PHQ, BSI, WHOQoL-Bref, FBeK)	Not suspected
Beisert et al., 2015 [20]	Age-, place of living-, and education-matched controls	Uncertainty present (diagnostic algorithm not reported)	Unclear (number of invited MRKH patients is not reported due to applied recruitement strategy)	Probably validated tools (Structured Clinical Interview “Psychosexual biorgaphy”)	Not suspected
Fliegner et al., 2013 [11]	Unclear (Statistical comparisons between MRKH and control group were not reported; baseline characteristics of control group were retrieved from a previously published paper)	Uncertainty present (diagnostic algorithm not reported)	Unclear (number of invited MRKH patients is not reported due to applied recruitement strategy)	Only validated tools (FUSS, RSES, FSFI)	Suspected, because the study was embedded in a larger project involving the same group of patients
Zhu et al., 2013 [22]	Age-matched controls	Uncertainty present (diagnostic algorithm not reported)	24 included out of 53 invited (54.7% loss of follow-up)	Only validated tools (FSFI)	Not suspected
Liao et al., 2011 [10]	Significant differences are highly suspected (Standarization samples used as control groups; different control group per outcome, details for control groups not reported)	Uncertainty present (diagnostic algorithm not reported)	56 or 36 patients included depending on outcome out of 93 invited (39.8% or 61,3% loss of follow-up)	Only validated tools (HADS, FSFI, MSQ, SF-12)	Not suspected
Csermely et al., 2011 [26]	Sexually active, age-matchedcontrols	None (diagnosis confirmed during laparoscopicVecchietti vaginoplasty)	23 patients included out of 23 invited (0% loss of follow-up)	Only validated tools (FSFI)	Not suspected
Kimberley et al., 2010 [27]	Australian population average as published in the WHOQoL User Manual served as control group; thus, control group includes males and has broader age range	Little uncertainty present (diagnosis confirmed by at leats two physicians)	28 patients included out of 61 invited (54.1% loss of follow-up)	Only validated tools (WHOQoL-Bref)	Not suspected; however, results for the Golombok and Rust Inventory of Sexual Satisfaction (GRISS) questionnaire were not compared to any control group and are not discussed in this systematic review
Gatti et al., 2010 [15]	Geographical location-matched controls who underwent surgery for minor pathological conditions at the same outpatient clinic	None (diagnosis confirmed during total vaginal replacement with sigmoid colon)	Unclear (number of invited MRKH patients is not reported)	Only validated tools (RSES, BDI, CTLMA)	Not suspected
Heller-Boersma et al., 2009 [8]	Sociodemographic characteristics may differ between MRKH and control group	None (women from the MRKH register at the U.K. National Centre for Adolescent and Adult Women with Congenital Abnormalities of the Genital Tract)	66 patients included out of 335 invited (80.3% loss of follow-up)	Only validated tools (SCL-90, RSES, IIP–32, EDI)	Not suspected
Laggari et al., 2009 [12]	Age- and school grade-matched, healthy, eumenorrheic, adolescent controls	Uncertainty present (diagnostic algorithm not reported)	5 patients included out of 5 invited (0% loss of follow-up)	Only validated tools (BDI, STAI)	Not suspected
Liu et al., 2009 [21]	Randomly selected, age-matched controls	None (diagnosis confirmed during laparoscopic Davydov vaginoplasty)	24 patients included out of 31 invited (22.6% loss of follow-up)	Only validated tools (FSFI)	Not suspected
Fedele et al., 2007 [4]	Age-matched controls	None	27 patients included out of 110 invited (75.5% loss of follow-up)	Only validated tools (FSFI)	Not suspected
Nadarajah et al., 2005 [24]	Significant differences (e.g. the average age of the control group was 39.7 years and 59.5% had children, whereas the average ae of the MRKH group was only 26.6, and 100% were nulliparous)	Uncertainty present (diagnostic algorithm not reported)	60 patients included out of 145 invited (58.6% loss of follow-up)	Only validated tools (FSFI)	Not suspected
Kaloo et al., 2002 [28]	The normal female Finnish population aged 19 to 39years used as controls	None (diagnosis confirmed during Laparoscopic-assisted Vecchietti procedure)	5 patients included out of 6 invited (16.7% loss of follow-up)	Only validated tools (Sintonen 15D questionnaire) and unvalidated tools (semi-structured telephone)	Not suspected
Raboch et al., 1982 [16]	Age-matched controls	None (diagnosis confirmed intraoperatively)	Unclear (number of invited MRKH patients is not reported)	Only validated tools (HTDW, SFW, SAI, Questionnaire N5)	Not suspected
